# Endobronchial Ultrasound Reliably Quantifies Airway Smooth Muscle Remodeling in an Equine Asthma Model

**DOI:** 10.1371/journal.pone.0136284

**Published:** 2015-09-08

**Authors:** Michela Bullone, Guy Beauchamp, Mireille Godbout, James G. Martin, Jean-Pierre Lavoie

**Affiliations:** 1 Department of Clinical Sciences, Faculty of Veterinary Medicine, Université de Montréal, St-Hyacinthe, Quebec, Canada; 2 Meakins-Christie Laboratories, Department of Medicine, McGill University, Montreal, Quebec, Canada; Georgia State University, UNITED STATES

## Abstract

Endobronchial ultrasonography (EBUS) revealed differences in the thickness of the layer representing subepithelial tissues (L2) between human asthmatics and controls, but whether this measurement correlates with airway smooth muscle (ASM) remodeling in asthma is unknown. In this study, we sought to determine the ability of EBUS to predict histological ASM remodeling in normal and equine asthmatic airways. We studied 109 isolated bronchi from the lungs of 13 horses. They underwent EBUS examination using a 30 MHz radial probe before being processed for histology. ASM remodeling parameters were evaluated in EBUS images (L2 thickness, L2 area, L2 area/internal perimeter [Pi] and L2 area/Pi^2^) and histological cuts (ASM area/Pi^2^), and compared. EBUS was then performed *ex vivo* on the lungs of 4 horses with heaves, an asthma-like condition of horses, and 7 controls to determine whether central bronchial remodeling could be detected with this technique. An optimized approach was developed based on data variability within airways, subjects, and groups, and then validated in 7 horses (3 controls, 4 with heaves) that underwent EBUS *in vivo*. L2 area was significantly associated to ASM area in isolated lungs (p<0.0001), in the absence of significant bias related to the airway size. Bronchial size significantly affected EBUS ASM-related parameters, except for L2 area/Pi^2^. L2 area/Pi^2^ was increased in the airways of asthmatic horses compared to controls, both *ex vivo* and *in vivo* (p<0.05). Bronchial histology confirmed our findings (A_ASM_/Pi^2^ was increased in asthmatic horses compared to controls, p<0.05). In both horses with heaves and controls, L2 was composed of ASM for the outer 75% of its thickness and by ECM for the remaining inner 25%. In conclusion, EBUS reliably allows assessment of asthma-associated ASM remodeling of central airways in a non-invasive way.

## Introduction

Airway smooth muscle (ASM) mass and phenotype as well as the amount and composition of extracellular matrix (ECM) mass are increased or altered in asthma [1]. Both large and small airways may suffer remodeling, the magnitude of which has been associated with disease severity [2–4]. Most human studies demonstrating remodeling of airway tissues have been performed on *post-mortem* specimens, especially when assessing peripheral airways or the most abaxial structures of the bronchial wall, such as ASM [5, 6]. Bronchoscopic biopsies may be valuable tools, but potentially suffer from issues of sampling [7]. There is a need for non-invasive methods to assess the airway wall structure *in vivo*, especially when assessing treatment-associated airway remodeling reversibility in asthma [4, 8].

Endobronchial ultrasonography (EBUS) is a non-invasive addition to routine bronchoscopy. Using radial mini-probes, the whole bronchial wall may be imaged and most of its structural components identified [9–11]. Two studies have shown that EBUS remodeling parameters such as the thickness of the first and second US layer (L1 and L2, representing epithelial and subepithelial tissue thickness including smooth muscle, respectively) differed between asthmatics and healthy patients [12], and also that they correlated with lung function [12, 13]. Increased thickness of the EBUS layer representing ASM and a variable portion of ECM (L2) was associated with greater values of basal membrane thickness [12]. However, whether EBUS allows for the specific assessment of ASM mass and its remodeling in asthma have not been demonstrated.

We investigated the correlation between ultrasonographic and histomorphometric measurement of ASM in in an spontaneously occurring asthma-like disease of horses (heaves) [14]. We hypothesized that heaves-affected horses sustain large airway remodeling compared to control subjects. Our objectives were to establish the comparability of measurements of airway wall structures assessed by EBUS to histology, to develop an optimized protocol for assessing remodeling of subepithelial structures in central equine airways *in vivo*.

## Methods

### Study design

The study was conducted in 5 consecutive phases, as summarized in Table 1. First, needle-puncture experiments were performed to describe the echographic anatomy of the laminar structures composing the equine bronchial wall. Then, isolated bronchial specimens were studied for direct comparison between EBUS and histology. *Ex vivo* examinations of equine lungs were performed as a “proof of concept” and for optimization of the technique used. *In vivo* EBUS examinations were then performed on a limited number of horses, subsequently euthanized in order to confirm our findings.

**Table 1:** Study design.

Phase	Aim(s)	Methods	Samples/Animals
1	Describing equine echographic bronchial anatomy	NPE	26 bronchial samples from lungs of 6 slaughtered horses*
2	Comparing measures of interest performed with EBUS and histology	EBUS and histology	109 bronchial samples from lungs of 13 slaughtered horses*
3	*Ex vivo* technique optimization and “proof of concept”	EBUS on isolated lungs	lungs from 4 horses with heaves and 7 controls
4	*In vivo* EBUS	EBUS on horses	4 horses with heaves and 3 controls
5	Validation a. assessing the effect of lung removal from the thorax (collapse) on EBUS; b. histological validation.	EBUS and histology on post-mortem specimens of horses studied in phase 4	same horses studied in phase 4

* Lungs with obvious macroscopic alterations were excluded from the study. NPE: needle puncture experiments; EBUS: endobronchial ultrasound.

### Animals

Seven horses underwent *in vivo* EBUS examination at the Veterinary Teaching Hospital (CHUV) of the Université de Montréal before being euthanized for reasons unrelated to respiratory diseases. Horses were humanely euthanized by intravenous injection of pentobarbital sodium. Available details of these animals are reported in Table 2 (clinical scores assessed as defined by Robinson [15] and septum thickness as defined by Koblinger [16]). All procedures described on living animals were performed in accordance with the guidelines of the Canadian Council on Animal Care and approved by the Animal Ethics Committee of the Université de Montréal (Rech-1663)(S1 Table reports the ARRIVE checklist).

**Table 2:** Details of the animals studied in *in vivo*.

Horse	Group	Age [years]	Breed	Disease status	Clinical score	Weight [kg]	Mucus score	Septum thickness	Bronchiectasis	DeltaP_L_ [cmH_2_O]	R_L_ [cmH_2_O/L/sec]
1	Control	12	QH	-	2/8	480	0/5	2/4	no		
2	Control	25	QH	-	3/8	450	0/5	0/4	regional		
3	Control	8	Pony	-	2/8	253	0/5	1/4	no		
4	Heaves	27	QH	Remission	4/8	438	1/5	2/4	yes		
5	Heaves	14	STB	Symptomatic	6/8	430	1/5	3/4	yes	25	1.8
6	Heaves	15	QH	Symptomatic	5/8	450	2/5	4/4	yes		
7	Heaves	27	Polo Pony	Symptomatic	6/8	450	3/5	2/4	regional		

QH: Quarter Horse; STB: Standardbred. Clinical scores were assessed as defined by Robinson [15]. Septum thickness was assessed as defined by Koblinger [16]. *Ante mortem* BAL data were not available. Pulmonary mechanics were available only for one horse, as it belonged to our research herd.

### Isolated lungs and bronchial tissues

Isolated lungs and bronchial tissues studied in phase 1, phase 2 and phase 3 of our work were obtained at a local slaughterhouse (Viande Richelieu Meat Inc, Massueville, QC, Canada) or from autopsy samples of horses euthanized at the CHUV of the Université de Montréal for reasons unrelated to respiratory diseases. The owners of the horses gave their consent for their animals to be used for this study. The lungs studied in phase 3 were obtained from 3 of the 4 horses with heaves and from 1 of the 7 healthy horses studied in the *in vivo* experiment.

### Equipment

All EBUS materials and equipment were purchased from Olympus (Richmond Hill, ON, Canada). Scans were obtained with a 30 MHz radial mini-probe (UM-S30-25R) connected to a dedicated ultrasound unit (EU-ME1). For *in vivo* EBUS, probes were inserted within a balloon-ended sheath (MH-246R) and passed through the working channel of a videoendoscope (CF-H260DL/I). EBUS experiments were recorded using iMovie on a MacBook Pro computer.

### EBUS procedure

*In vivo* bronchoscopies were performed under sedation (detomidine 0.02 mg/kg and butorphanol 0.01 mg/kg injected IV) and topical anesthesia (lidocaine solution 0.5%). The videoendoscope was inserted through a nostril and advanced until it wedged within the main caudal lobar bronchus. Images were collected from all bronchial segments within reach, up to the main bronchi. *Ex vivo* examinations were similarly performed on lungs kept on ice until examined (<6 hours following euthanasia). One lung per animal was examined. Lungs showing macroscopic alterations were excluded.

### EBUS image acquisition

Videos were analyzed at slow motion and good quality EBUS images were saved as tiff files. Quality of EBUS scans was assessed on the basis of resolution and definition of the layered structure, as well as on uniformity and completeness of the acquired scan.

### Needle Puncture Experiments

**Fig 1:**
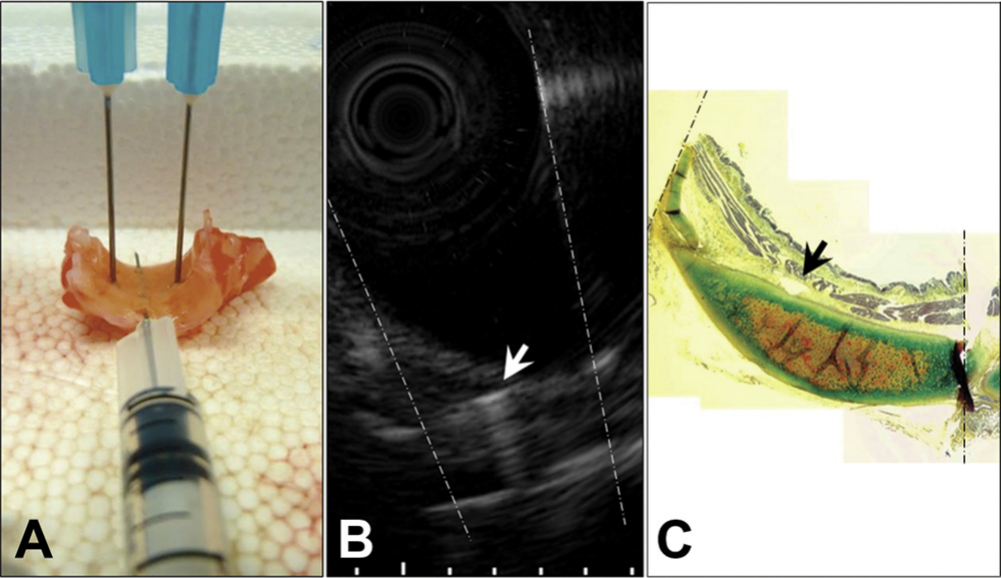
Illustrative images of a needle puncture experiment (NPE). Note the echo produced by the needle inserted longitudinally to the long axis of the bronchus in B (white arrow), and its corresponding histological localization as identified in C (black arrow). Dotted lines represent needles positioned transversally to the longitudinal axis of the bronchus.

Bronchial sections ~3-mm long and ~10-mm wide were randomly collected from the lungs. Needle-puncture experiments were performed as previously described [11]. Briefly, samples were flattened and fixed with two 18G needles to a polystyrene surface (Fig 1A); a third smaller needle (27G) was then inserted longitudinally into the airway wall under stereoscopic microscope guidance. With the samples submerged in saline solution, EBUS images were captured when all needles were visible (Fig 1B). Airways were then fixed and paraffin embedded with the needles kept in place in order to reproduce histological cuts similar to the EBUS views. The layer in which the needle was identified in EBUS images was compared to the histological structure in which it was found at histology (Fig 1C).

### Isolated bronchial specimen analysis (EBUS vs. histology)

**Fig 2:**
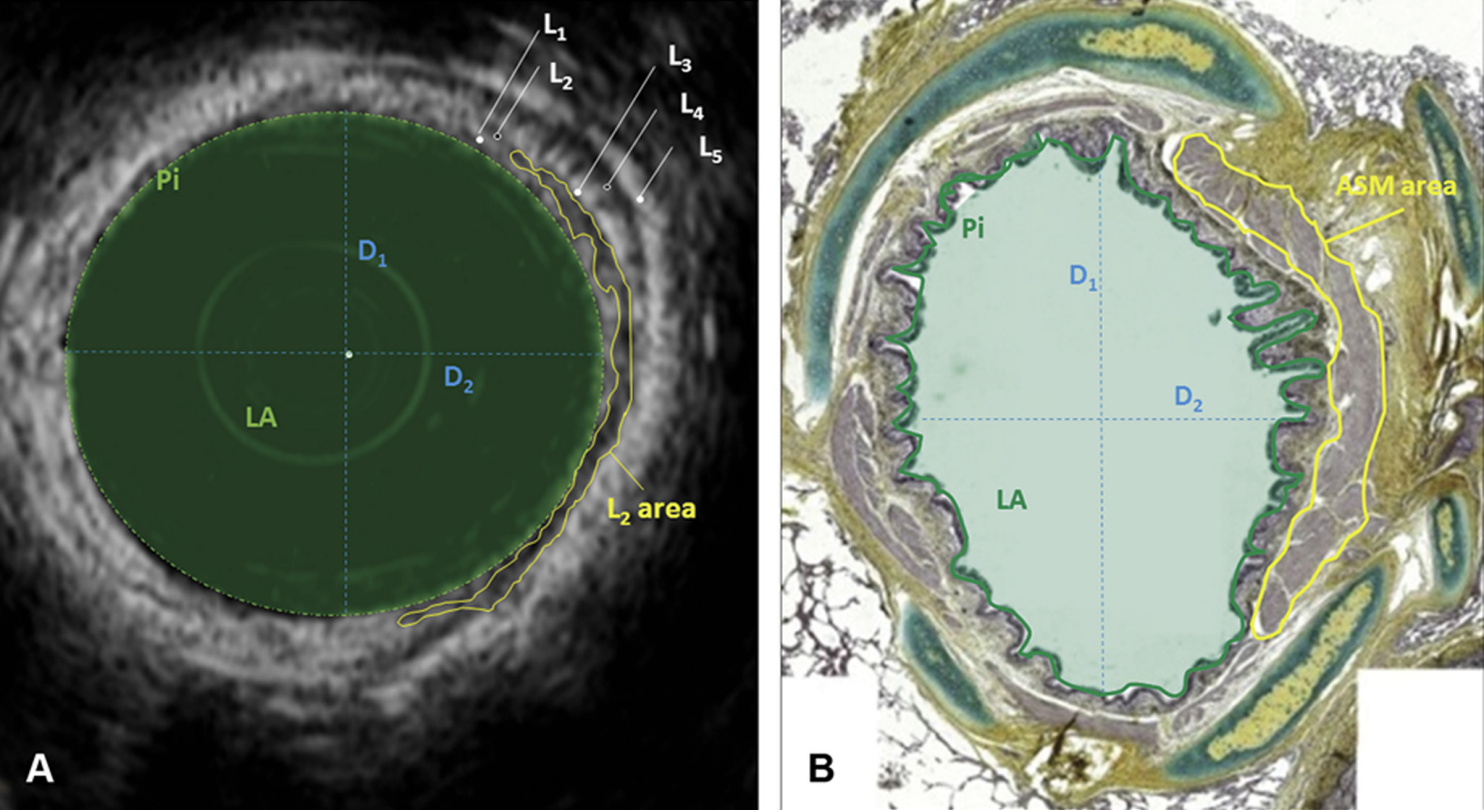
Measurements made on EBUS (A) and histological (B) images. Only a part of L2 area and ASM area have been encircled in yellow, to allow the reader appreciate the rest of the image. L: US layer; D_1_ and D_2_: perpendicular diameters (blue dotted lines); LA: lumen area (filled light green area); Pi: airway perimeter (continuous green line).

Eight to 10 complete bronchial sections were randomly chosen and carefully dissected from each lung. Bronchi were immersed in saline solution and EBUS images acquired before processing for histology. The following measurements were made: bronchial diameter (D, calculated as the mean of 2 perpendicular diameters measured in each airway), internal perimeter (Pi), lumen area (LA), thickness of the first and second layer (L1 and L2, respectively, calculated as the mean of 5 values measured at predetermined sites) as well as the area of the second layer (L2 area). Images were excluded from analyses if the bronchial wall was not clearly identifiable for >180° (missing angle). L2 area/Pi and L2 area/Pi^2^ ratios were calculated. After formalin fixation and paraffin embedding, sections of 5-μm thickness were obtained, stained with Movat's pentachrome and digitized at 2.5-5x magnification, using a Leica camera (DCF320, Leica Microsystems, Cambridge, UK) and Photoshop CS5 (Adobe Systems Inc., San Jose, CA, USA). Histological images were scored from 1 to 4 (S2 Table), based on tissue architecture preservation and perpendicularity of the cut. Images scored 1 were excluded from analysis. Images scored 2 were considered only if the missing angle was <90°. For all airways, the mean of 2 diameters (D), internal perimeter (Pi), lumen area (LA) and ASM area (A_ASM_) were measured (Fig 2), and A_ASM_/Pi^2^ ratio calculated. All measurements were made blindly using ImageJ (version 1.48c, NIH, Bethesda, MD, USA). When images were incomplete, the subtended angle of the analyzed part was recorded and measurements were transformed into complete measurements proportionally. Fourteen EBUS images and 14 bronchial sections, selected randomly, were analyzed by the same observer three times at ≥ one week interval in order to assess repeatability of measures.

### Composition of L2

**Fig 3:**
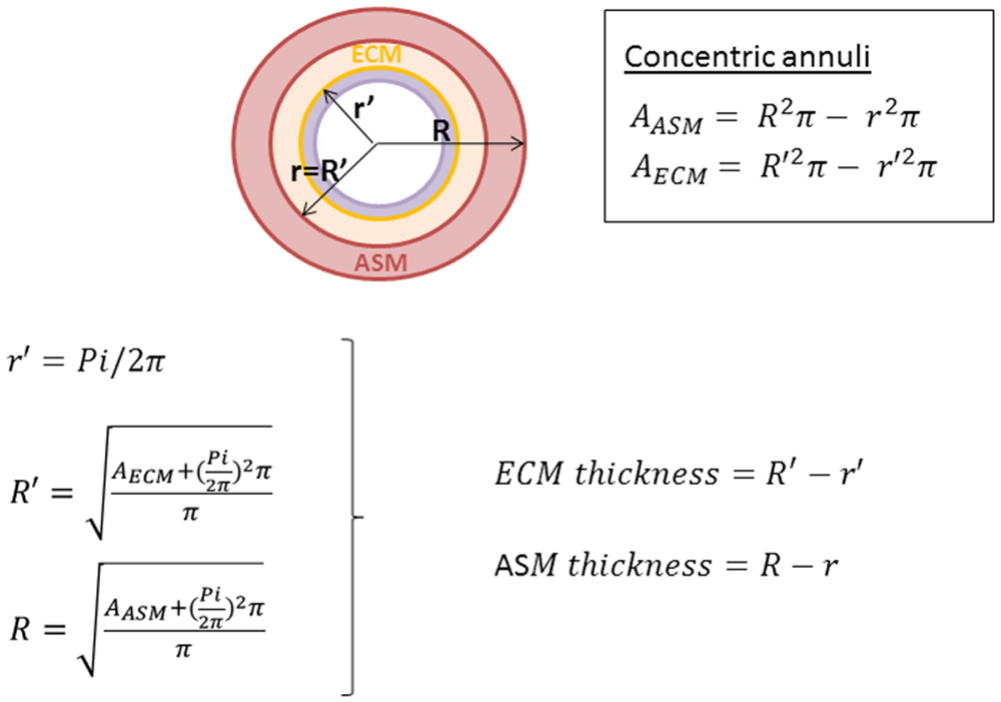
Scheme of a distended bronchial wall. In our mathematical model, the bronchial wall was treated as a structure composed of multiple concentric annuli. Excluding the inner annulus, representing the epithelial layer (in purple, negligible in thickness), we considered the submucosa as composed by two annuli representing the ECM (yellow) and ASM (pink). Pi corresponded to the yellow circumference in our model.

The composition of L2 was studied using the EBUS images obtained *in vivo* and the histological sections obtained at post-mortem from the same lungs. As airways are naturally distended *in vivo* during EBUS but they contract during the fixation and embedding processes necessary for histology, we developed a method to estimate the mean thicknesses of the ECM (lamina propria) and ASM layers for maximally dilated airways (Fig 3). In brief, airways were treated as a series of concentric annuli in which the thickness of the epithelial layer was considered as negligible. In our model, Pi values measured in histological sections corresponded to the circumference of the smaller circle of the inner annulus (representing ECM), and were used to calculate its radius (r’). The radius of the larger circle (R’) of the inner annulus was then calculated as a function of ECM area and Pi. The difference between the larger and the smaller radii of the inner annulus (R’-r’) corresponded to the ECM thickness. The same approach was used to calculate ASM thickness (R-r), with R’ being used as the smaller radius of the outer annulus (r). To validate our approach, the calculated ECM and ASM thicknesses were compared with values measured using standard histomorphometric techniques [3, 7, 17] on the same histological sections: the mean value of 5 measures performed randomly for each airway represented our measured ECM and ASM thickness.

Finally, the sum of ECM and ASM thickness calculated at histology (histologic submucosal thickness) was compared to the sum of L1 and L2 thickness (EBUS submucosal thickness). The percentage of the total airway thickness occupied by ECM and ASM at histology was calculated and related to the thickness and composition of L1 and L2.

### Statistical analysis

Statistical analyses were performed with SAS v.9.3 (Cary, NC, USA). Mixed linear models with “subject” used as a random effect, and Bland-Altman tests were used to compare measurements from EBUS and histology. Mixed linear models with “group” and “bronchial size” as fixed effects were used for group comparisons including random factors to take into account the non-independent measures from the same subject for different bronchial sizes and for repetitions within bronchial size. *A priori* contrasts were performed, adjusting the alpha level of each comparison with the Bonferroni sequential procedure, to compare group means at each bronchial size. Two types of airways were defined depending on the size of Pi (intermediate: Pi ≤31 mm; large: Pi>31 mm). Mixed covariance linear models were used for studying how variables varied with Pi; the “group” was used as a fixed effect, “Pi” was used as a co-factor and “subject” as a random effect.

Variance component analyses were performed for the repeatability of EBUS and bronchial section measurements. The same analysis was also carried out within individuals for repeated measurements of L1, L2, L2 area/Pi and L2 area/Pi^2^. The effect of bronchial size (3 classes, based on Pi: 0–16 mm, 16–31 mm and >31 mm) was studied with a mixed linear model with “group” and “Pi class” as fixed effects. *A priori* contrasts were performed, again with the sequential Bonferroni procedure, to compare group means within each Pi class. The coefficient of variation (CV) was calculated for airway values obtained from 2, 3, and 4 images, to determine how many images should be analyzed to provide a reliable estimate. The level of statistical significance was set at 0.05 throughout.

## Results

### Phase 1—Needle Puncture Experiments

**Fig 4:**
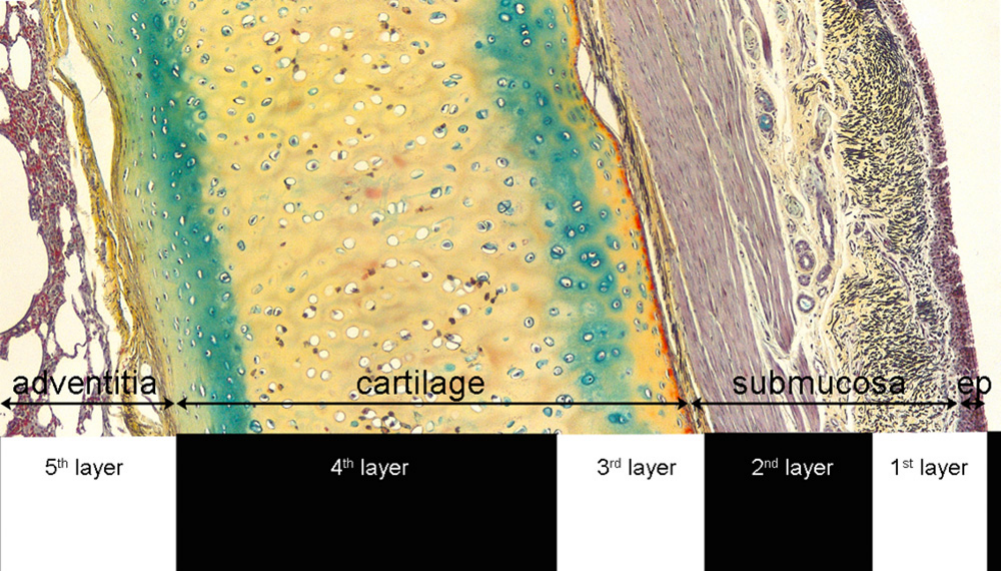
Comparison of EBUS and histological composition of equine airways. In absence of cartilages, L3 was represented by the adventitia.

Experiments were performed on 26 bronchial samples from 6 horses. The first hyperechoic layer (L1) corresponds to the epithelium and to a variable portion of the underlying extracellular matrix (ECM). The second hypoechoic layer (L2) corresponds to the remaining fraction of ECM and smooth muscle (Fig 4). When cartilage is present, L3, L4 and L5 correspond to the cartilage internal surface, cartilage thickness, and external cartilage surface and adventitia, respectively. Occasionally, a second, more abaxial cartilage could be identified. In the absence of cartilage, L3 corresponds to the adventitia.

### Phase 2—EBUS-histology comparison

A total of 109 isolated bronchi obtained from 13 horses were scanned and subsequently processed for histology. Repeatability of histologic measurements was excellent with an intra-class correlation coefficient of 99.0% for Pi, 98.5% for A_ASM_, and 95.5% for A_ASM_/Pi^2^ values. Repeatability of EBUS measurements was also generally good with an intraclass correlation coefficient (ICC) of 99.6% for Pi, 81.6% for L1, 87.3% for L2, 94.2% for L2 area/Pi, and 92.3% for L2 area/Pi^2^ values. Overall, a good agreement was observed between the two methods for all parameters measured, except for L2 area/Pi^2^ and A_ASM_/Pi^2^ (see data in Table 3 and S1 Fig). Bland-Altman tests revealed that EBUS tended to underestimate Pi when compared to histology, and differences tended to get larger as values of Pi increased (S2 Fig). Also, EBUS overestimated D values for small bronchi and underestimated them for large bronchi. However, these differences in D were considered clinically negligible, as the mean difference between the 2 measurements was 0.14 mm.

**Table 3:** Mixed linear models and Bland-Altman tests for comparison between EBUS and histologic measurements.

		Mixed linear model		Bland-Altman test (EBUS-histology)				
Parameter	N	p-value	R^2^	Difference mean (SD)	Mean mean (SD)	Slope^1^	Intercept^2^	Systematic bias
Perimeter [mm]	101	<0.0001	0.20	-5.04 (6.99)	22.67 (8.52)	-0.42, p<0.0001	4.46, p = 0.02	yes
Diameter [mm]	101	<0.0001	0.30	0.14 (1.51)	4.7 (2.15)	-0.15, p = 0.04	0.87, p = 0.03	yes
Lumen area [mm^2^]	21	<0.0001	0.80	-4.59 (15.98)	34.07 (42.67)	-0.15, p = 0.08	0.44, p<0.98	no
A_ASM_*L2 area [mm^2^]	98	<0.0001	0.31	-0.0534 (1.843)	3.3263 (2.99)	-0.01, p = 0.083	0.005, p<0.99	no
A_ASM_/Pi*L2 area/Pi [mm]	98	0.001	0.16	0.0312 (0.0634)	0.1323 (0.06)	0.27, p = 0.02	-0.004, p<0.78	yes
A_ASM_/Pi^2^*L2 area/Pi^2^ [–]	98	0.45	0.030	0.0029 (0.0038)	0.0062 (0.002)	0.93, p<0.0001	-0.003, p = 0.006	yes

^1^ p values refer to the null hypothesis that the slope = 0.

^2^ p values refer to the null hypothesis that the intercept = 0.

### Phase 3—*Ex vivo* test and optimization

**Fig 5:**
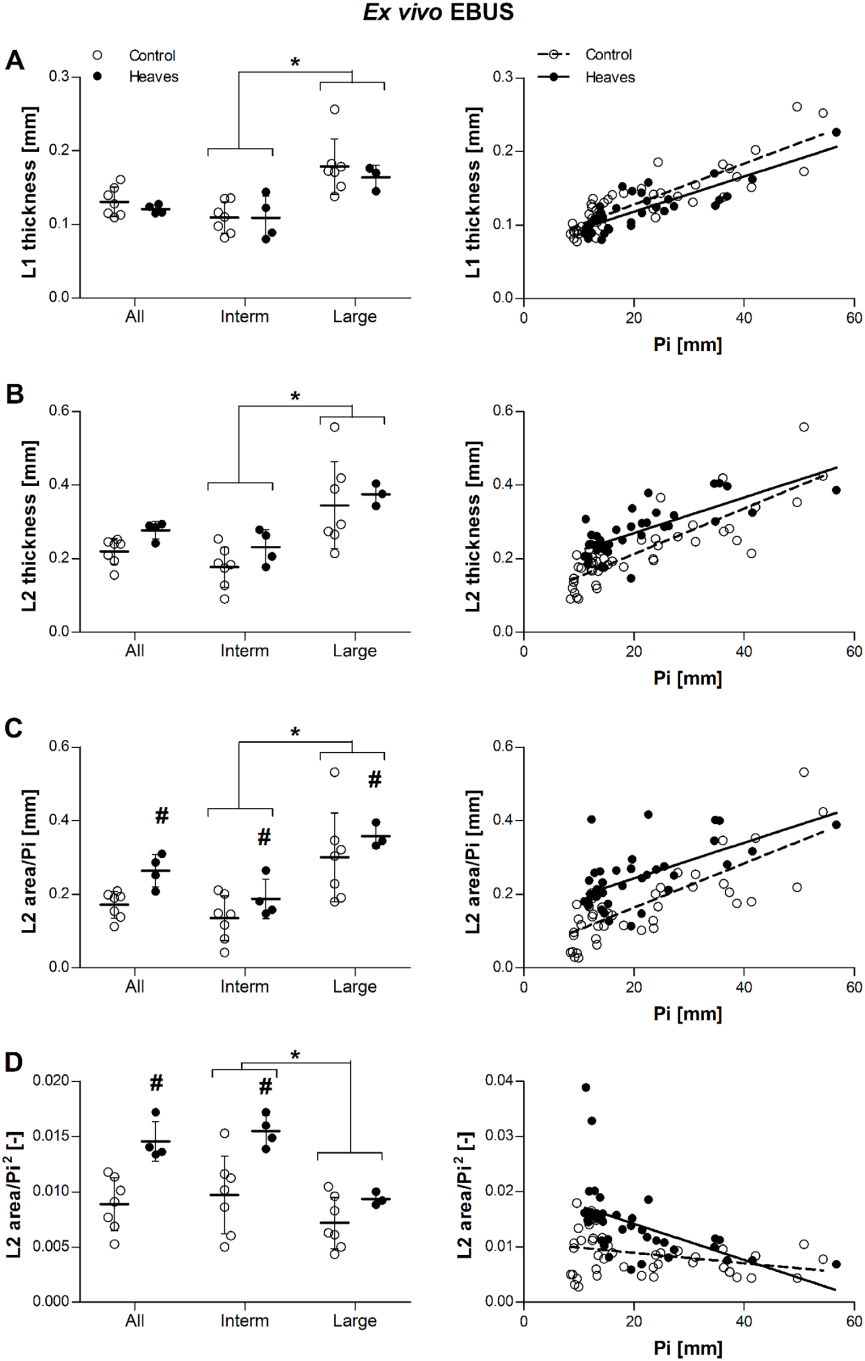
Effect of group and airway size on L1 thickness (A), L2 thickness (B), ratio L2 area/Pi (C), and ratio L2 area/Pi^2^ (D) in *ex vivo* EBUS. Raw data (before statistical corrections) are presented as mean per animal in the graphs on the left, and as mean per airway the graphs on the right. L1 and L2 thickness was similar in horses with heaves and controls across all bronchi (p = 0.4 and p = 0.1, respectively). Contrarily, L2 area/Pi and L2 area/Pi^2^ ratios were greater in horses with heaves than controls across all bronchi (p = 0.004 and p = 0.02, respectively). L1 values were similar in intermediate (Pi<31mm, p = 0.82) and large bronchi (Pi>31mm, p = 0.32) analyzed separately. L2 tended to be thicker in intermediate bronchi of horses with heaves than in controls (p = 0.06) but not in large bronchi (p = 0.5). Both L2 area/Pi and L2 area/Pi^2^ ratios were greater in intermediate bronchi of horses with heaves then controls (p = 0.006 and p = 0.003, respectively), but only L2 area/Pi ratios were greater also in large bronchi (p = 0.04; p = 0.3 for L2 area/Pi^2^ ratios). Overall, L1, L2 and L2 area/Pi values were greater in large than intermediate bronchi (p = 0.0003, p = 0.001, and p = 0.002, respectively), while L2 area/Pi^2^ values were greater in intermediate than in large bronchi (p = 0.02). L1, L2, and L2 area/Pi increased significantly with Pi in both groups (p<0.0001 for all), while L2 area/Pi^2^ decreased significantly with increasing Pi (p<0.0001). The slope of the relationship did not differ between the two groups for L1, L2 and L2 area/Pi (p = 1, p = 0.14, p = 0.66, respectively), but it was significantly greater in the heaves group than in controls for L2 area/Pi^2^ (p = 0.01). #: significantly different from controls. *: significant difference between values of intermediate and large bronchi (pooling the two groups).

Four lungs obtained from horses with heaves and 7 from controls were studied after removal from the thoracic cage. None of the lungs showed significant macroscopic alterations. A mean of 8 to 10 airways was analyzed in each lung. Our first aim was to investigate whether a difference in submucosal remodeling could be observed *ex vivo* between horses with heaves and controls, in the absence of any noise generated by respiratory movements. Airways of similar sizes were analyzed in both groups (Pi [mean±SD] 20.6±4.9mm in heaves; 21.2±3.5mm in controls, p = 0.65). When bronchi of both groups of horses were analyzed together, values of L1, (p = 0.0003), L2 (p = 0.001), and L2 area/Pi (p = 0.004) were significantly larger in large rather than intermediate airways. Conversely, L2 area/Pi^2^ was larger in intermediate rather than in large airways (p = 0.02), which we ascribe to the effect of the disease (remodeling). In both groups of horses, L1, L2 and L2 area/Pi increased with Pi, while L2 area/Pi^2^ decreased (statistical details are provided in Fig 5). When analyzing only intermediate airways, the slope of the curve of the control group for L2 area/Pi^2^ was not significantly different from zero (slope = -0.00022, p = 0.13). The slope of the relationship between L2 area/Pi^2^ and Pi was significantly higher in the heaves group than in the control group (p = 0.01). L2 (p = 0.009), L2 area/Pi (p = 0.004) and L2 area/Pi^2^ (p = 0.02), but not L1 (p = 0.4) values, were significantly larger in horses with heaves than in the controls. L2 area/Pi (p = 0.006) and L2 area/Pi^2^ (p = 0.003) were also greater in intermediate airways of horses with heaves than in controls. L2 tended to be thicker in intermediate bronchi of horses with heaves than controls, although it did not reach statistical significance (p = 0.06). In large bronchi, only L2 area/Pi values were greater in horses with heaves than controls (p = 0.006) (Fig 5).

Secondly, we aimed at optimizing our imaging technique using an approach as similar as possible to those we use *in vivo*. The greatest variation observed in all EBUS parameters measured *ex vivo* (L1, L2, L2 area/Pi and L2 area/Pi^2^) was attributable to the size of the airways in individual horses. However, analyzing 8–10 airways per horse of similar mean size allowed yielding significantly higher inter-group variation compared to inter-subject variation within the same group (Table 4). CV analysis showed that a minimum of 3 images of good quality for each airway provided a reliable estimate of the real values of all the parameters analyzed.

**Table 4:** Variance component analysis.

**Ex vivo analysis**				
Parameter	Variation (%)			
	Group	Subject	Airway	Images
L1	0	8.5	**72.2**	19.3
L2	27.1	3.3	**55.7**	13.9
L2 area/Pi	38.4	5.1	**41.5**	15
L2 area/Pi^2^	32.5	0	**42.5**	25
**In vivo analysis**				
Parameter	Variation (%)			
	Group	Subject	Airway	Images
L1	7.2	12.6	**53.6**	26.6
L2	33.6	0	**43.1**	23.3
L2 area/Pi	**46.3**	0	30.2	23.5
L2 area/Pi^2^	29.7	7.4	10.3	**52.6**

### Phases 4 and 5—*In vivo* EBUS and histological validation

EBUS was performed *in vivo* in 7 animals (4 horses with heaves and 3 controls), all of which were then euthanized and EBUS immediately repeated on 4 of them (3 heaves and 1 control, as the lungs of the other animals were severely contaminated with blood). EBUS was performed on the same lung imaged *in vivo* using the same approach, in order to compare the results. A similar number of airways was analyzed on the two occasions (10.7±4/horse *in vivo* and 9.5±4/horse *ex vivo*, p = 0.6). Three to 4 images were analyzed for each airway. Lung collapse occurring with lung removal from the thoracic cage did not significantly change the values of L2, L2 area/Pi, and L2 area/Pi^2^ (p = 0.46, p = 0.79 and p = 0.17, respectively). L1 tended to be thicker when measured *ex vivo* (p = 0.09), however. This supports and validates the *ex vivo* findings we have described above.

**Fig 6:**
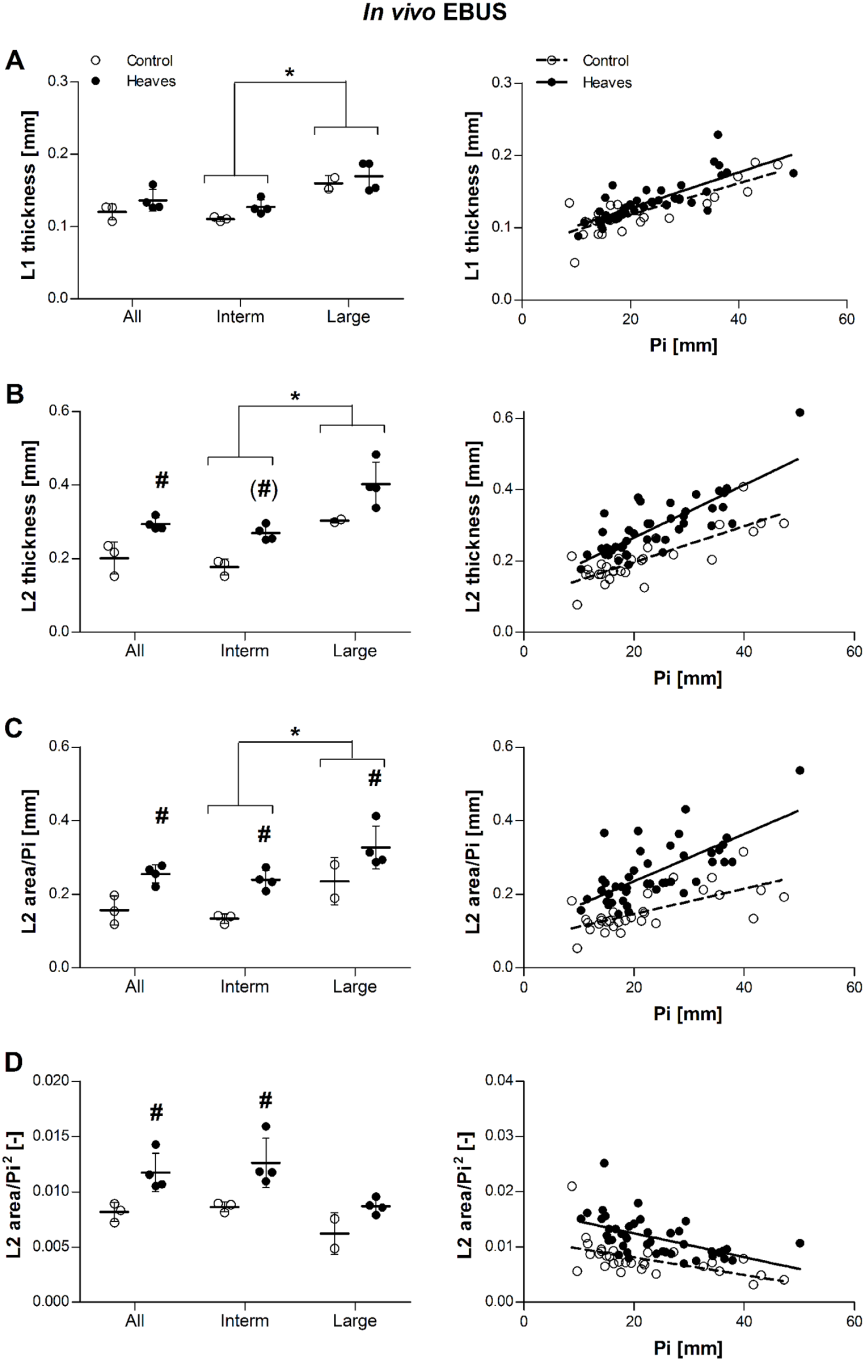
Effect of group and airway size on L1 thickness (A), L2 thickness (B), ratio L2 area/Pi (C), and ratio L2 area/Pi2 (D) in *in vivo* EBUS. Raw data (before statistical corrections) are presented as mean per animal in the graphs on the left, and as mean per airway the graphs on the right. L1 and was similar in horses with heaves and controls across all bronchi (p = 0.27). Contrarily, L2 thickness, L2 area/Pi and L2 area/Pi^2^ ratios were greater in horses with heaves than controls across all bronchi (p = 0.03, p = 0.005 and p = 0.04, respectively). L1 values were similar in intermediate (Pi<31mm, p = 0.13) and large bronchi (Pi>31mm, p = 0.63) analyzed separately. L2 tended to be thicker in intermediate bronchi of horses with heaves than in controls (p = 0.03, non-significant after correction) but not in large bronchi (p = 0.13). Both L2 area/Pi and L2 area/Pi^2^ ratios were greater in intermediate bronchi of horses with heaves then controls (p = 0.003 and p = 0.02, respectively), but only L2 area/Pi ratios were greater also in large bronchi (p = 0.04; p = 0.22 for L2 area/Pi^2^ ratios). Overall, L1, L2 and L2 area/Pi values were greater in large than intermediate bronchi (p = 0.002, p = 0.005, and p = 0.01, respectively), while L2 area/Pi^2^ only tended to be greater in intermediate than in large bronchi (p = 0.05). L1, L2, and L2 area/Pi increased significantly with Pi in both groups (p<0.001 for all), while L2 area/Pi^2^ decreased significantly with increasing Pi (p<0.001). The slope of the relationship did not differ between the two groups for L1 and L2 area/Pi^2^ (p = 0.86 and p = 0.49, respectively), and it was significantly greater for L2 and L2 area/Pi (p = 0.02 and p = 0.04, respectively). Filled dots represent heaves and open dots represent controls. #: significantly different from controls. *: significant difference between values of intermediate and large bronchi (pooling the two groups).

*In vivo*, in both groups of horses, L1, L2 and L2 area/Pi increased with Pi, while L2 area/Pi^2^ decreased (statistical details are provided in the legend of Fig 6). As previously shown *ex vivo*, the slope of the relationship between L2 area/Pi^2^ and Pi was not statistically different from zero in intermediate airways of control horses (slope = -0.00026, p = 0.08). The slopes of the relationships between L2 and Pi, and between L2 area/Pi and Pi were significantly higher in horses with heaves than controls (p = 0.02 and p = 0.04, respectively). L2 (p = 0.03), L2 area/Pi (p = 0.005), and L2 area/Pi^2^ (p = 0.04), but not L1 (p = 0.27) values, were significantly larger in horses with heaves than in controls. L2 area/Pi was significantly larger in intermediate airways of horses with heaves than in controls (p = 0.003). L2 and L2 area/Pi^2^ values tended to be greater in intermediate airways of horses with heaves than in controls (p = 0.03 and p = 0.02, respectively, but not statistically significant after Bonferroni correction). In large bronchi, only L2 area/Pi values were higher in horses with heaves than controls (p = 0.04) (Fig 6C). Power analysis indicated that n = 6 horses per group would be needed to show a statistically significant effect 80% of the times if there was a 30% difference of L2 thickness or a 60% difference of L2 area/Pi^2^ between the two groups when n≈10 bronchi of similar size are analyzed in each subject (Table 5).

**Table 5:** Power analysis for *in vivo* EBUS.

Parameter	Expected magnitude of the effect	N
*L2*	15%	22
	30%	**6**
	60%	2
*L2 area/Pi*	15%	80
	30%	21
	60%	**7**
*L2 area/Pi* ^*2*^	15%	76
	30%	21
	60%	**6**

N represents the number of subjects needed to obtain a statistically significant effect of the group 80% of the times for different effect sizes (expressed as %) using the methodology described earlier.

**Fig 7:**
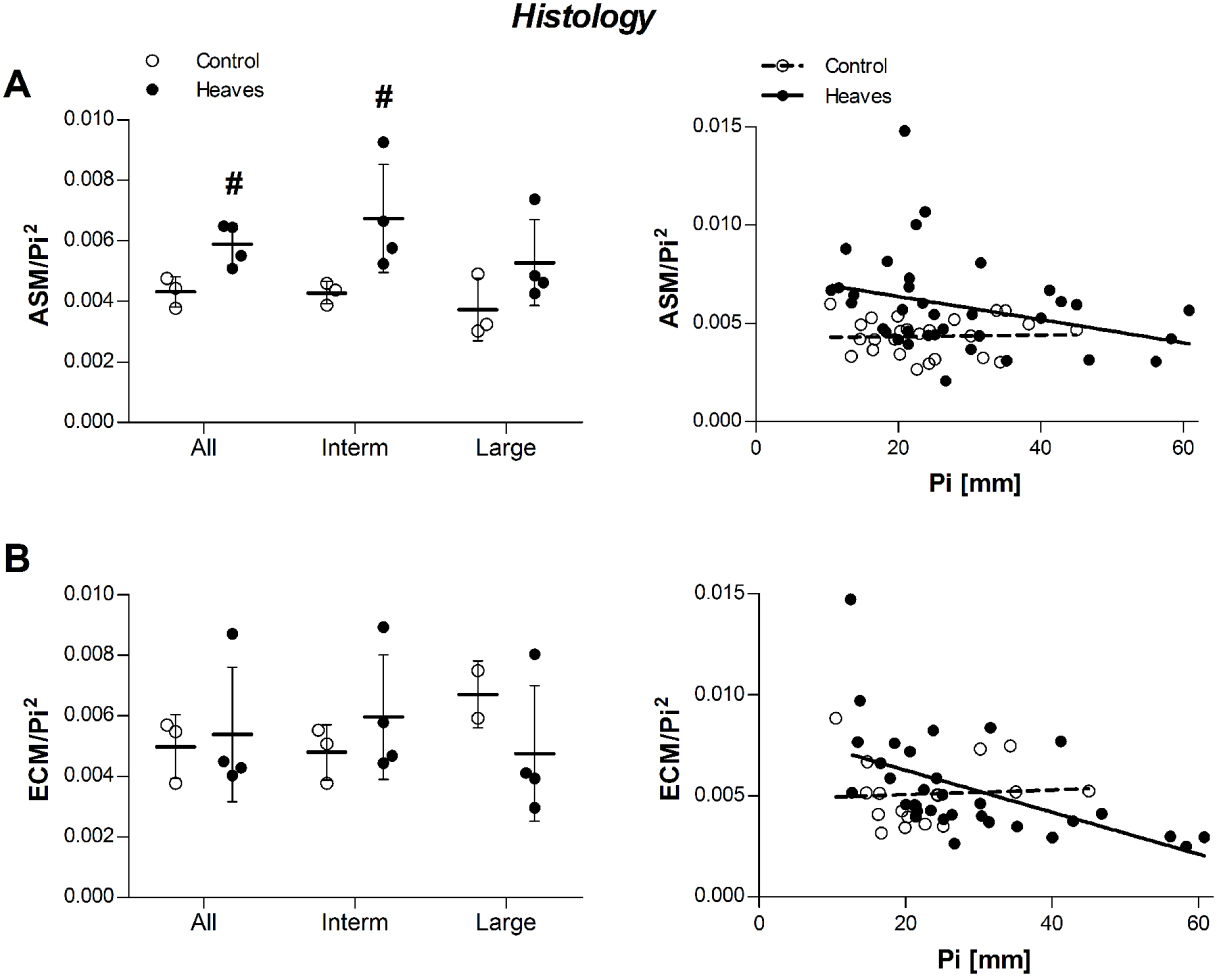
Effect of group and airway size on A_ASM_/Pi^2^ (A) and A_ECM_/Pi^2^ (B) measured on histological sections. Raw data (before statistical corrections) are presented as mean per animal in the graphs on the left, and as mean per airway the graphs on the right. Histological analysis confirmed *in vivo* and *ex vivo* EBUS findings concerning ASM remodeling. The slope of the A_ASM_/Pi^2^ relationship was similar between the 2 groups (p = 0.26), but heaves-affected horses had significantly higher values compared to controls (p = 0.04). The slope of the A_ECM_/Pi^2^ relationship tended to be different between the 2 groups (p = 0.07), but heaves-affected horses had similar values compared to controls (p = 0.26). #: significantly different from controls. *: significant difference between values of intermediate and large bronchi (pooling the two groups).

*Post-mortem* histological analyses were performed. Morphometrical analysis of 8 to 10 bronchial sections per lung confirmed that A_ASM_/Pi^2^ was significantly larger in horses with heaves than in the controls (p = 0.04) pooling all bronchi. Overall, A_ASM_/Pi^2^ values were larger in intermediate than in large bronchi (p = 0.02) and a significant difference between horses with heaves and controls was observed in intermediate (p = 0.03) but not in large bronchi (p = 0.29). A_ECM_/Pi^2^ values were similar between the two groups across all bronchi (p = 0.26) and also when measured in intermediate and large bronchi separately (p>0.1 for both, Fig 7).

### Composition of L2

**Fig 8:**
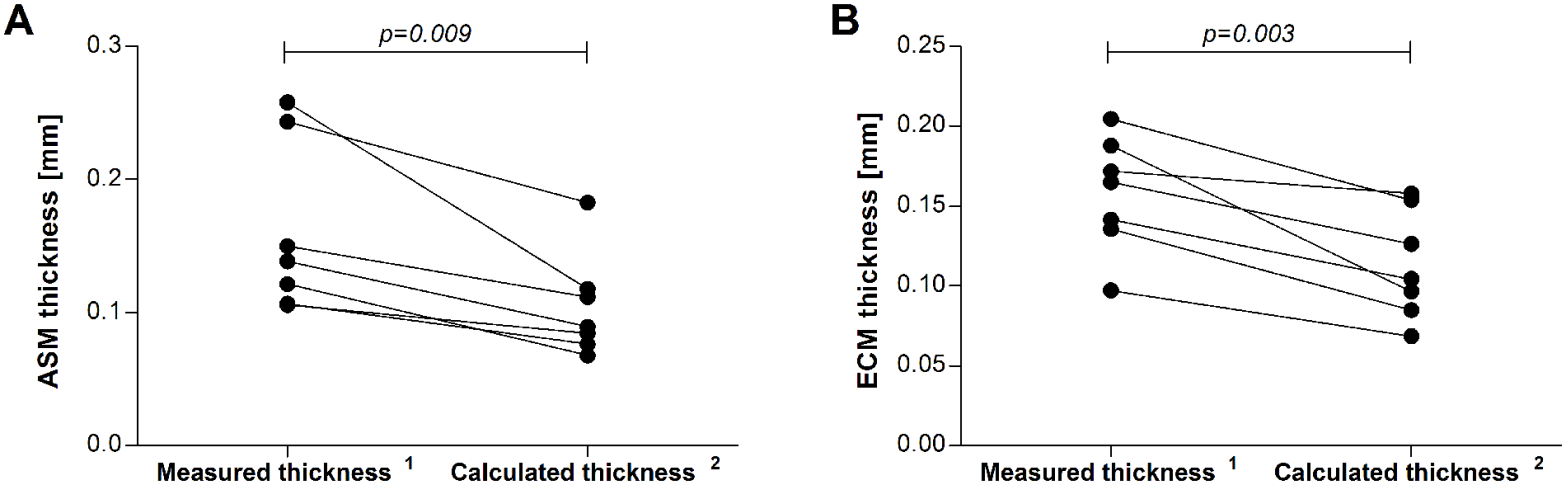
Effect of the method used for measuring ASM (A) and ECM (B) thickness in histologic bronchial samples. Results are shown as mean values per horse. ^1^Mean value of 5 measures of ASM or ECM thickness performed manually and randomly around the bronchial circumference (ECM was measured from the basal membrane to the ASM inner border, avoiding regions where obvious collagen fiber shredding occurred). ^2^Thickness of the ASM or ECM calculated as function of the ASM or ECM area and airway Pi, as if it was a continuous and homogeneous layer for an airway completely distended (Pi corresponds to the inner circumference of an annulus). Statistical differences were calculated with paired t-tests.

**Fig 9:**
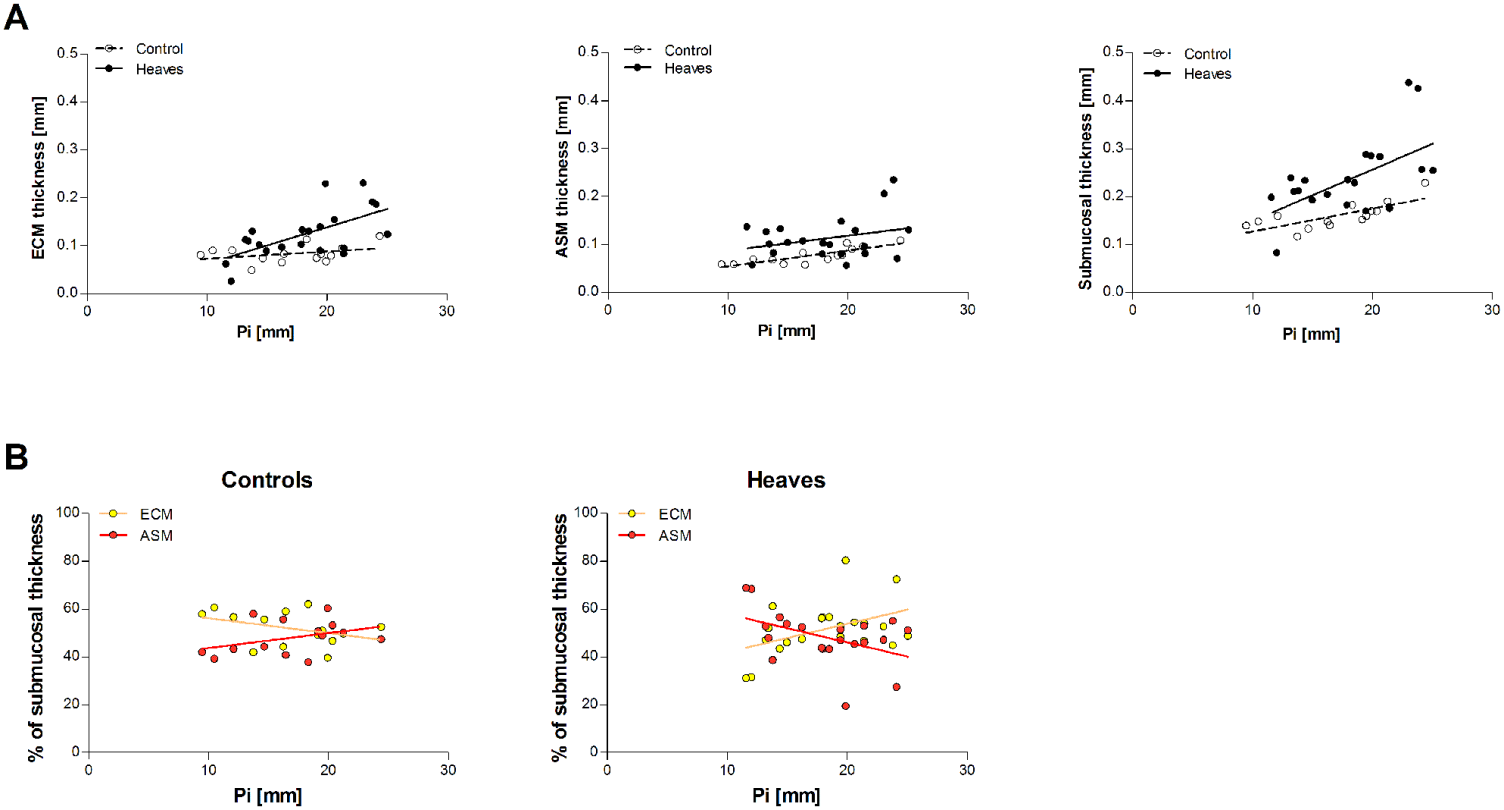
Composition of the submucosal tissues at histology. Values are presented as percentage of the submucosal tissue occupied by ECM and ASM for each airway. N = 14 for controls and 20 for heaves. On average, a greater percentage of submucosal thickness was occupied by ASM in smaller airways of horses with heaves compared to controls. This tendency reverses as the airway size increases. However, as the submucosal thickness is increased in horses with heaves compared to controls, the absolute thickness of the ASM layer still remains greater in horses with heaves than in controls for airways with Pi<26mm.

**Fig 10:**
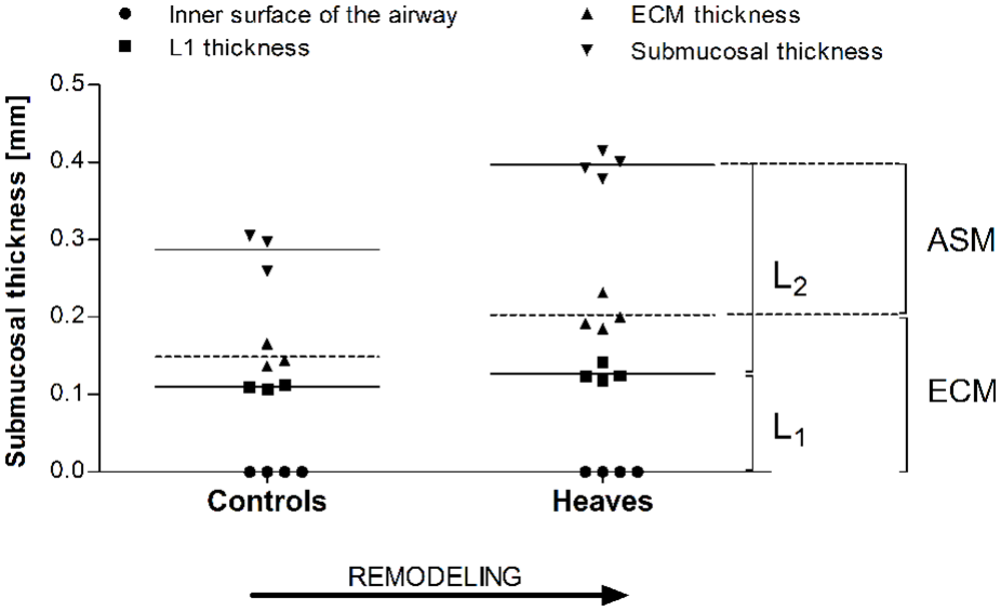
Composition of L2 is similar in asthmatic and control horses. The inner 25% is occupied by ECM while the remaining outer 75% is occupied by ASM. Notice that most of the ECM thickness lies behind the echo of L1.

The mean thickness of ASM in the airways of both heaves-affected and control horses varied with airway size (p = 0.0009). For this reason, we analyzed only airways with Pi<26mm, whose number and mean Pi was similar in both groups (Table 6). ASM and ECM calculated thickness were increased in the airways of asthmatic horses compared to controls (p = 0.057 and p = 0.02, respectively). Interestingly, both ASM and ECM thickness were overestimated when measured manually compared to when it was calculated using our derived measurements (mean±SD reduction of 33.3±12.1% for ASM and of 28.5±12.5% for ECM calculated values, Fig 8). Histologic submucosal thickness (the sum of ECM and ASM calculated thicknesses) was increased in horses with heaves compared to controls (p = 0.027). The submucosal composition of the equine airways is affected by both disease and airway size (Fig 9). Indeed, the slopes of ECM% and ASM% were significantly different in the 2 groups (p = 0.02) and a significant association between Pi and ECM% and between Pi and ASM% was found in horses with heaves (p = 0.04) but not in controls (p = 0.2). No significant differences were identified between the mean proportional content of ECM or ASM relative to the total submucosal layer between controls and heaves-affected horses (p = 0.3 for both). L1 represented entirely ECM in horses of both groups. L2 represented ECM and ASM in similar proportion in both groups of horses. Indeed, on average, 22% of L2 represented ECM and 78% represented the ASM in control horses, while 28% of L2 represented ECM and 72% represented ASM in horses with heaves (p = 0.6 for both) (Fig 10).

**Table 6:** Data used for determining L2 composition.

	Controls	Heaves	*p value*
**Histology**			
N [airways/horse]	4.67±0.57	5±2.16	0.58
Pi [mm]	18.94±1.91	20.09±3.93	1.00
ECM thickness [mm]	0.0833±0.014	0.1356±0.025	**0.02**
ASM thickness [mm]	0.0762±0.008	0.1254±0.04	**0.057**
Submucosal thickness [mm]	0.160±0.011	0.261±0.05	**0.027**
Submucosal ECM % at histology	52±3.2	53.5±2.9	0.3
Submucosal ASM % at histology	48±3.2	46.5±2.9	0.3
**EBUS**			
N [airways/horse]	7.67±5303	8.5±3.70	0.85
Pi [mm]	16.19±1.71	19.86±1.61	0.11
L1 thickness [mm]	0.11±0.003	0.127±0.010	0.06
L2 thickness [mm]	0.177±0.022	0.269±0.021	**0.004**
Submucosal thickness [mm]	0.287±0.024	0.397±0.015	**0.024**
Submucosal L1% at EBUS	38.5±2.5	32.1±3.2	**0.037**
Submucosal L2% at EBUS	61.5±2.5	67.9±3.2	**0.037**
**Composition of L1**			
ECM%	100	100	1
ASM%	0	0	1
**Composition of L2**			
ECM%	22.1±8.9	28.1±5.7	0.63
ASM%	77.9±8.9	71.9±5.7	0.63
**Others**			
Shrinkage (%)	44.28±4.88	47.41±7.92	0.7

Values are presented as mean±SD of the values obtained from the airways of 4 horses with heaves and 3 controls. Bold characters indicate significance as results of t-tests. Mean values per horse were used as statistical unit.

## Discussion

Airway remodeling is a central feature of asthma. ASM mass is increased in central airways, more prominently in severe and fatal asthma [2]. However, its quantification *in vivo* is difficult, as imaging techniques such as computerized tomography or magnetic resonance imaging do not allow visualization of ASM, and endobronchial biopsies often provide incomplete sampling [8]. While EBUS has revealed an increase in L2 thickness in asthmatics compared to healthy subjects, which has been linked to ASM remodeling indirectly by means of bronchoprovocation tests [13], this is the first report directly comparing L2 remodeling at EBUS and ASM remodeling at the level of histology. Using EBUS, we found that in the central airways of horses L2 is composed mainly by ASM (about 75% of its thickness) with the remaining 25% representing ECM, independently from the presence or absence of heaves-associated remodeling. Indeed, EBUS reliably quantified the ASM mass in both healthy and asthmatic animals, providing accurate estimates compared to histology, and this despite the high variation in remodeling likely to occur even within the same airway as reported in man [18]. Our *in vivo* results support EBUS employment for monitoring bronchial remodeling over time or during pharmacological treatments in this model. Importantly, its relative non-invasiveness will permit evaluating the same sites at different points in time.

Echographic anatomy of the equine bronchial wall is similar to that of man [11, 13] and sheep [19], in which this technique has previously been reported. The thickness of the first EBUS layer (L1), representing the bronchial epithelium and the most axial part of the underlying ECM, was not affected by disease status in our study. Contrarily, the thickness of the second EBUS layer (L2), representing the more abaxial part of the ECM and the ASM, was significantly increased in horses with heaves compared to controls. Curiously, L2 (and not L1) thickness has been shown to correlate with basal membrane thickness in human asthma although a significant difference between the thickness of L1 was observed in the same study between asthmatics and controls [12]. As L2 represents ASM and a variable part of the ECM [11] which does not include the basal membrane, its significant relationship with basal membrane thickness in asthmatics was more likely a spurious correlation rather than a histologic confirmation of EBUS findings. Indeed, it has been shown that the reticular basement membrane thickness of central airways is correlated with submucosal airway remodeling in cartilaginous airways of asthmatics [8]. Increased basal membrane thickness has not been reported in heaves to our knowledge. However, preliminary observations from our laboratory suggest that basal membrane thickening is only an occasional finding in heaves, validating the fact that no difference was observed in L1 thickness between the 2 groups that we studied. Our results highlighted that ASM was mostly responsible for the increased L2 thickness in EBUS images of asthmatic horses compared to controls, and demonstrated that not only small [20, 21] but also large airways can sustain remodeling in heaves. The significant association observed between values of A_ASM_ and L2 area, the absence of systematic bias for ASM quantification between histology and EBUS, and the similarity of ECM mass in the 2 groups found in this study already suggested that L2 changes in asthmatic horses mainly reflected ASM mass increases. This was confirmed by the finding that ASM represents on average 75% of L2 in both control and asthmatic horses. Horses experiencing exacerbations of the disease showed increased ECM mass in endobronchial biopsies (measured as the distance between the epithelium and the ASM layer) compared to controls [7]. In our study, we also found that ECM calculated thickness was increased in intermediate bronchi of asthmatic horses compared to controls. However, values of A_ECM_/Pi^2^ were similar in both groups. As both ASM and ECM thickness increase at increasing Pi, it is possible that despite Pi of the airways analyzed in both groups being similar, the small gap between the mean values of airway size of the 2 groups could have accounted for the statistical significance. Alternatively, as ECM represents constantly 25% of L2 and L2 thickness is increased in heaves, it is likely that ECM sustains a certain degree of remodeling in heaves, but it could be mild and thus lost with normalization (correction by Pi^2^). Finally, analyses for ECM/Pi^2^ were made on intermediate airways with Pi<31mm while analyses for ECM thickness were made on airways with Pi<26 mm, in order to have the same number of airways of similar Pi in both groups. It is possible that values of airways with 26mm<Pi<31mm could have accounted for the difference observed.

We have attempted for the first time to describe the composition of L2 in the asthmatic airways by studying naturally occurring equine heaves. Our results indicate that ASM accounts for approximately 75% of L2 in both healthy and diseased animals, with ECM representing the remaining 25%. Our finding of a constant portion of L2 being occupied by ASM is in agreement with the results of a previous study in which a significant correlation between L2 thickness and airway hyperresponsiveness was found in asthmatic patients [13]. Indeed, two different studies have shown that the increase in ASM mass is potentially the most important structural determinant explaining hyperresponsiveness in asthmatics [22, 23]. Data obtained on lungs of healthy and asthmatic human lungs suggest that the proportion of the submucosa occupied by the ASM in man might be lower compared to those we found in horses in airways of similar size [5, 24]. Nevertheless, the thickness of the submucosal tissues measured at EBUS appears to be similar between the two species, considering airways of similar size [12].

We have also explored for the first time the relationship between bronchial size and ASM remodeling as estimated by EBUS (L2 thickness or L2 area). L2 thickness increased linearly with bronchial size in both asthmatic and healthy equine airways, which is in agreement with the ASM distribution along the bronchial tree (expressed as ASM thickness) in man [25]. Remarkably, such increase was steeper in asthmatic airways compared to controls. This finding, supported by our histological findings, indicates that heaves can display a phenotype similar to the type II form of asthma as defined by Ebina [24], characterized by remodeling of small [26] and large airways, at least in some cases. We also explored L2 area/Pi^2^ ratio, as its histological equivalent A_ASM_/Pi^2^ is commonly used for ASM mass normalization [26, 27]. Pi^2^ accounts for the relatively greater amount of both ASM and ECM components in small than in large bronchi [28, 29]. As previously demonstrated for A_ASM_/Pi^2^, the slope of the curve of L2 area/Pi^2^ ratio plotted against airway size (expressed as Pi) was non-significant in intermediate airways of control horses, confirming appropriateness of normalization. The lack of correlation between L2 area/Pi^2^ ratio and its histological equivalent A_ASM_/Pi^2^ was possibly due to amplification of the bias of Pi (underestimated by EBUS, especially in larger airways) when raised to the power of 2. However, since bias of Pi measures was constant in both groups, we judged it correct to compare their L2 area/Pi^2^ ratio values in our analysis.

Variability among different airways constituted the main source of variation in our *ex vivo* study for the remodeling parameters assessed. This finding was expected due to our study design, in which airways of different size were studied in each subject. However, the second greatest source of variation was represented by the group (heaves vs control) for all parameters studied, supporting the role of EBUS in the assessment of central remodeling in heaves. Similar findings were observed *in vivo*. A greater variation among different images of the same airway was observed *in vivo* compared *to ex vivo*, possibly due to the respiratory movements. L2 area/Pi^2^ variation among different airways was greater *ex vivo* than *in vivo*. We ascribed this finding to the fact that different degrees of pulmonary collapse could have occurred in the lungs of different animals or at different levels of the bronchial tree within the same lungs. The finding that L1, L2, and L2 area/Pi are affected by airway size highlights the importance of sampling airways of similar sizes in different subjects in order to avoid sampling bias when these parameters are used.

From a clinical perspective, our results support the implementation of EBUS as a tool for assessing large airway remodeling in asthmatic patients. Despite its relatively low invasiveness (compared for instance to the endobronchial biopsy procedure) and relative ease of analysis of measurements, this technique is underemployed in asthma possibly because of its slow learning curve [30, 31], and paucity of data supporting its use to assess airway remodeling in asthma. Our findings that EBUS reliably assesses ASM mass in proximal airways in heaves, combined with the previous report correlating EBUS L2 thickness and lung function in asthma [13], support the use of this technique for evaluating the efficacy of treatments at reducing ASM mass asthmatics. EBUS may also prove useful for defining specific asthma phenotypes based on different ASM remodeling patterns along the bronchial tree [32], possibly predicting their response to specific treatments. In this perspective, the assessment of a single airway as described in previous studies investigating airway remodeling with EBUS [12, 13] does not take into account the heterogeneity existing among different airways or even among different sections of the same airway [18], and could represent an important source of bias even when the same airway is evaluated in all subjects. Our study showed that the assessment of 8 to 10 bronchi reliably estimated the ASM mass in both heathy and asthmatic horses, and it was always performed in less than 15 minutes, which do not exceed times reported in man for a standard EBUS procedure [33].

Our study has some limitations. First, we could not obtain physiological data of the horses studied *in vivo*. Indeed, most horses undergoing euthanasia were client-owned, and the short period of time elapsing between euthanasia decision and death did not allow lung function testing. The number of horses employed in the *in vivo* phase is limited, but as *in vivo* data reflected what previously observed *ex vivo* we believe that it was sufficient to support our conclusions. Finally, the morphometric analyses performed on ECM were limited to the lamina propria, as we did not consider the ECM elements outside the ASM layer. This was mainly due to technical difficulties, as collagen fiber shredding often preventing a reliable assessment of ECM mass outside the ASM layer. Previous studies evaluating ECM remodeling in horses with heaves and asthmatic patients have used the same approach both in small and in large airways [3, 7, 17, 21].

In conclusion, our results indicate that EBUS provides reliable estimates of ASM remodeling in both asthmatic and healthy airways, and allows differentiation of such conditions based on remodeling features. Contrarily to endobronchial biopsies, it offers the possibility of studying non-carinae-derived bronchial tissue and to do it prospectively in the same airways as physical removal of bronchial tissue is not required. Finally, EBUS represent a promising technique for assessment of drug efficiency in reversing asthma-associated remodeling over time in a safe and non-invasive way.

## Supporting Information

S1 Fig Reliability of EBUS compared to histology (mixed lineal model). Results of mixed linear models for the association between the measures of perimeter (A), diameter (D), lumen area (E), ASM area vs L2 area (B), ASM area/Pi versus L2/Pi (C) and ASM/Pi^2^ versus L2 area/Pi^2^ (F) obtained with EBUS and histology. Each dot represents the mean of the measures made for a single airway.
(TIF)

S2 Fig Reliability of EBUS compared to histology (Bland-Altman tests). Comparison of measures of perimeter (A), ASM area (B), ASM area/Pi (C), diameter (D), lumen area (E) and ASM area/Pi^2^ (F) obtained with EBUS or histologic images studied with Bland-Altman tests. Each dot represents the mean of the measures made for a single airway. Continuous lines represent the mean difference between the two measuring methods (EBUS-histology). Dotted lines define the area where 95% of the differences should occur assuming a normal distribution of the differences. A negative difference means that EBUS values are smaller than the corresponding histology measurements.
(TIF)

S1 Table The ARRIVE Guidelines Checklist. (PDF)

S2 Table Histological quality score. Images with score 1, 2 and 3 were taken at 2.5x magnification, while image scored 4 was taken at 5x magnification (scale bars = 1 mm in all figures).
(PDF)
