# Supplementary figures and images for: Endobronchial Ultrasound Reliably Quantifies Airway Smooth Muscle Remodeling in an Equine Asthma Model
Source: PLoS One. 2015 Sep 8;10(9):e0136284. doi: 10.1371/journal.pone.0136284 (PMC4562526; doi:10.1371/journal.pone.0136284)

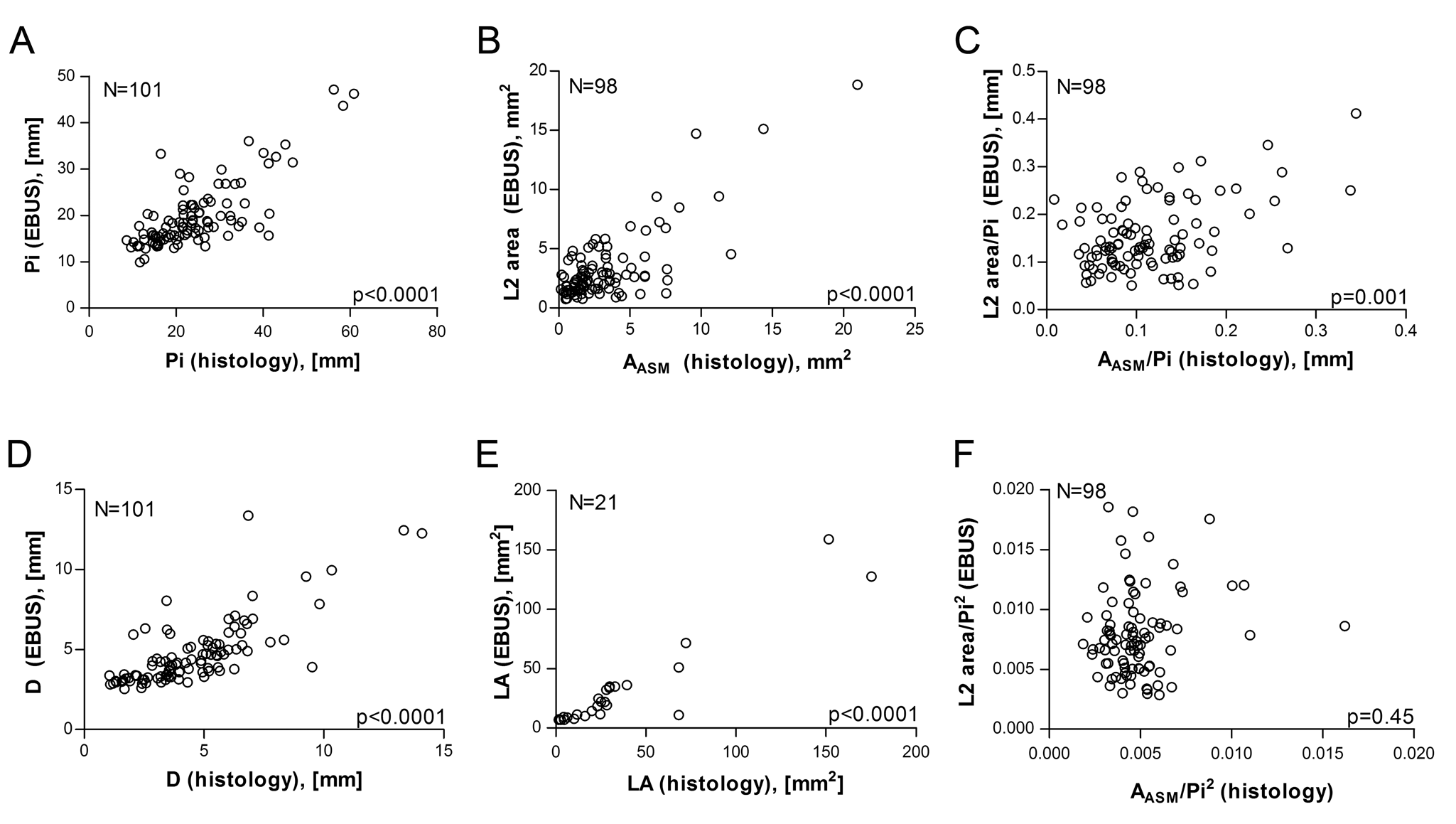

Supplement: S1 Fig — Results of mixed linear models for the association between the measures of perimeter (A), diameter (D), lumen area (E), ASM area vs L2 area (B), ASM area/Pi versus L2/Pi (C) and ASM/Pi2 versus L2 area/Pi2 (F) obtained with EBUS and histology. Each dot represents the mean of the measures made for a single airway. (TIF) [file pone.0136284.s001.tif]

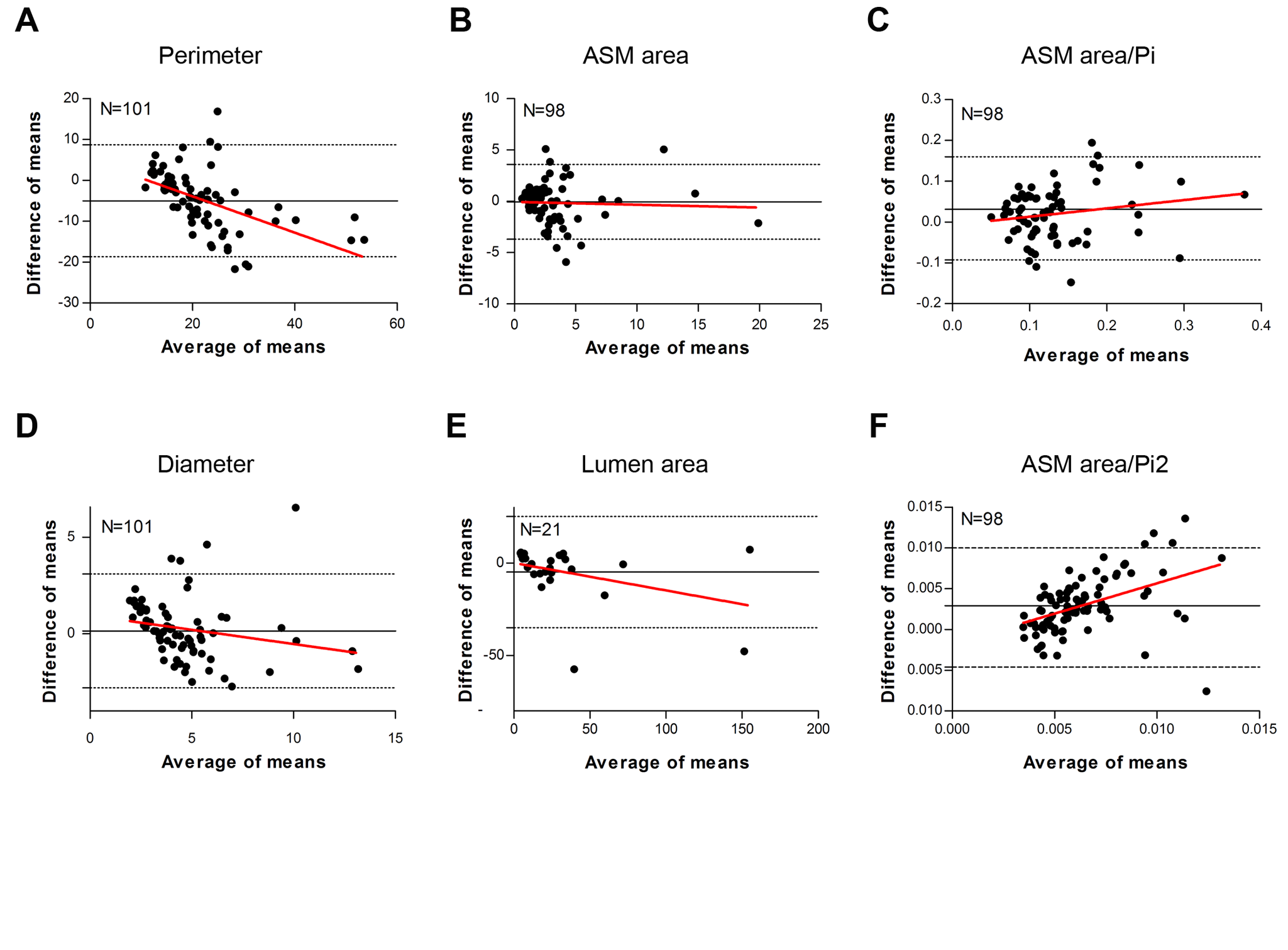

Supplement: S2 Fig — Comparison of measures of perimeter (A), ASM area (B), ASM area/Pi (C), diameter (D), lumen area (E) and ASM area/Pi2 (F) obtained with EBUS or histologic images studied with Bland-Altman tests. Each dot represents the mean of the measures made for a single airway. Continuous lines represent the mean difference between the two measuring methods (EBUS-histology). Dotted lines define the area where 95% of the differences should occur assuming a normal distribution of the differences. A negative difference means that EBUS values are smaller than the corresponding histology measurements. (TIF) [file pone.0136284.s002.tif]
