# Supplementary material for: Endobronchial Ultrasound Reliably Quantifies Airway Smooth Muscle Remodeling in an Equine Asthma Model
Source: PLoS One. 2015 Sep 8;10(9):e0136284. doi: 10.1371/journal.pone.0136284 (PMC4562526; doi:10.1371/journal.pone.0136284)
Supplement: S2 Table — Images with score 1, 2 and 3 were taken at 2.5x magnification, while image scored 4 was taken at 5x magnification (scale bars = 1 mm in all figures). (PDF) [file pone.0136284.s004.pdf]

Supplementary material

Airway section	Score	Description
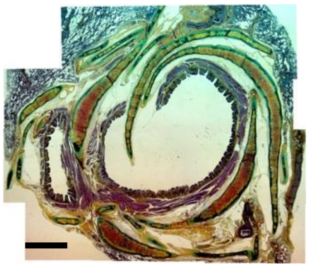	1	Airway wall missing for >90° or cut oblique to the perpendicular axis of the airway or significantly altered tissue architecture
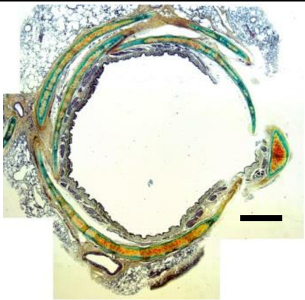	2	Cut perpendicular or slightly oblique to the airway longitudinal axis or airway wall missing for >45°, well preserved tissue architecture
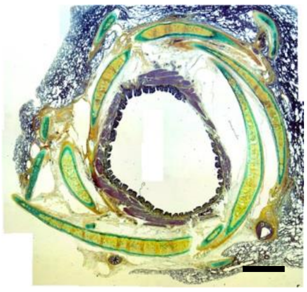	3	Cut perpendicular to the airway longitudinal axis, airway wall components (epithelium/ASM) missing for 15-45°, well preserved tissue architecture
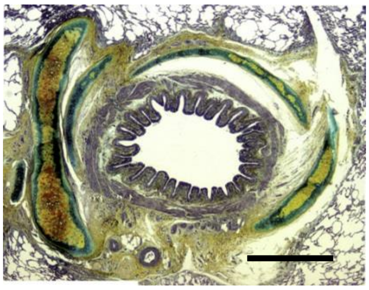	4	Cut perpendicular to the airway longitudinal axis, complete airway wall, very well preserved tissue architecture

Table S-I. Histological quality score. Images with score 1, 2 and 3 were taken at 2.5x magnification, while image scored 4 was taken at 5x magnification (scale bars = 1 mm in all figures).
